# Incidence of sudden cardiac death in adults with end-stage renal disease: a systematic review and meta-analysis

**DOI:** 10.1186/s12882-016-0293-8

**Published:** 2016-07-11

**Authors:** Sharanya Ramesh, Ann Zalucky, Brenda R. Hemmelgarn, Derek J. Roberts, Sofia B. Ahmed, Stephen B. Wilton, Min Jun

**Affiliations:** Cumming School of Medicine, University of Calgary, Calgary, AB Canada; Department of Medicine, Division of Nephrology, University of Calgary, Health Sciences Building, Room G233, 3330 Hospital Drive NW, Calgary, AB T2N 4N1 Canada; Department of Community Health Sciences, University of Calgary, Calgary, AB Canada; Department of Surgery, University of Calgary and the Foothills Medical Centre, Calgary, AB Canada; Libin Cardiovascular Institute of Alberta, Calgary, AB Canada; The George Institute for Global Health, The University of Sydney, Sydney, Australia

**Keywords:** Sudden cardiac death, End stage renal disease, Incidence, Systematic review

## Abstract

**Background:**

Although sudden cardiac death (SCD) is recognized as a distinct cause of death in patients with end stage renal disease (ESRD), its incidence has not been well summarized.

**Methods:**

We performed a systematic review and meta-analysis of the literature based on a protocol developed *a priori*. We searched MEDLINE and EMBASE (inception to March 2015) for randomized controlled trials and cohort studies reporting the incidence of SCD in adult patients with ESRD on hemodialysis or peritoneal dialysis. We collected data on number of SCD as well as the definition of SCD for each individual study. A random-effects model was used to summarize the incidence of SCD. We conducted subgroup analyses to explore sources of heterogeneity.

**Results:**

Forty two studies (*n* = 80,382 patients) were included in the meta-analysis. The incidence of SCD among adults with ESRD ranged from 0.4 to 10.04 deaths per 100 person-years. The definitions and assessment of SCD varied across the included studies. There was evidence of significant heterogeneity (I^2^ = 98; *p* < 0.001), which was not explained by subgroup analyses stratified by mean age, proportion of hypertensive or diabetic patients, follow-up time, study size, or type of cohort studied.

**Conclusion:**

Current estimates of the incidence of SCD among adults with ESRD vary widely. There is a need for further studies to more accurately estimate the incidence of SCD in patients with ESRD.

**Electronic supplementary material:**

The online version of this article (doi:10.1186/s12882-016-0293-8) contains supplementary material, which is available to authorized users.

## Background

The rising prevalence of end stage renal disease (ESRD) is a global public health concern [1–3]. Adults with ESRD have mortality rates up to 30-fold higher than the general population, with cardiovascular disease the major cause of death, accounting for approximately 38 % of all deaths among patients receiving chronic dialysis [4]. Sudden cardiac death (SCD), typically defined as death due to cardiac arrest occurring suddenly and within 1 h of witnessed symptom onset (or occurring within 24 h since last the patient had been known to be well), is responsible for the majority of cardiovascular-related deaths in patients with ESRD with studies reporting that up to 25 % of all deaths in this high-risk population is attributable to SCD [5]. However, there is substantial inconsistency in the definition of SCD, leading to wide variations in the reported SCD rates among individuals with ESRD [6]. Narrative reviews have attempted to summarize SCD rates in patients with ESRD [5, 7, 8], however, these reviews have not been systematically conducted, and their primary purpose was to summarize possible causes and mechanisms of SCD. We therefore sought to conduct a systematic review and meta-analysis of randomized trials and cohort studies to determine the incidence of SCD in adults with ESRD.

## Methods

### Data sources and searches

We performed a systematic review of the literature based on a protocol developed *a priori* in accordance with recommendations from the Meta-analysis of Observational Studies in Epidemiology and Preferred Reporting Items for Systematic Reviews and Meta-analyses statements [9].

We identified relevant studies by searching Ovid MEDLINE (from 1950 to March 2015) and EMBASE (from 1980 to March 2015) without language restrictions. All relevant text words and Medical Subject Heading (MeSH)/Emtree terms for chronic kidney disease (“Renal Insufficiency Chronic”, “Kidney Failure”, “Kidney Diseases”, “Renal Replacement Therapy”, “Uremia”, “Dialysis”, “Hemodialysis”, “Hemofiltration”, “Peritoneal Dialysis” or “Predialysis”) and SCD (“Heart Arrest” or “Sudden Cardiac Death” or “Sudden Arrhythmic Death”) were combined separately using the “OR” Boolean operator. These two search themes were then combined using the Boolean “AND” operator (Additional file 1). To identify additional relevant studies, we manually screened reference lists from identified studies and contacted field experts.

### Study selection

Two independent reviewers (SR and AZ) screened all abstracts identified by the search using a standardized approach to determine relevant articles for full-text review. All cohort studies and randomized controlled trials conducted in adults (age ≥18 years) with ESRD receiving dialysis (hemodialysis or peritoneal dialysis) and reporting on ≥5 events of SCD were eligible for inclusion in the systematic review. Studies examining the incidence of SCD in transplant recipients were excluded. Any disagreement in study inclusion was resolved by a third reviewer (MJ).

### Data extraction

A standardized data extraction spreadsheet was developed and used to abstract data on baseline participant characteristics including age, sex, body mass index, systolic and diastolic blood pressure, proportion of patients with diabetes, hypertension, myocardial infarction, coronary artery disease, current smokers, alcohol intake, and ethnicity, as well as follow-up duration, and SCD (as defined by authors).

### Study quality assessment

Study quality was judged based on standard criteria relevant to systematic reviews of cohort studies [10] and the Cochrane Risk of Bias for randomized controlled trials. For observational studies, the criteria assessed were patient attrition, acknowledgement of sources of funding, clear description of SCD, clear description of study participant characteristics, number of participants at each stage of study and reasons for ineligibility, proportion of eligible participants described, evidence of consecutive or random sampling, clear descriptions of inclusion criteria and sources and methods of participant selection and study setting, and locations and dates for recruitment. For randomized trials, study quality was judged by proper conduct of randomization, treatment allocation concealment, use of intention-to-treat, blinding of participants and outcome assessment, and evidence for incomplete or selective reporting of outcome. For each criterion, a study was considered to be at low risk if the information for the assessment was provided and there was sufficient evidence to fulfill criteria requirement. A study was considered at medium risk if there was evidence to suggest that part of the criteria requirement was completed, however, not sufficiently to reduce bias. A study was considered to be high risk if no information was provided or if no information regarding a failure to meet criteria requirements was provided.

### Outcome

We collected data on the number of SCD events as well as the definition of SCD for each individual study.

### Data synthesis and analysis

The incidence of SCD in each of the individual studies and the corresponding Wilson score 95 % confidence intervals (CIs) for binomial data were calculated [11]. In calculating the incidence, the total number of patients at risk was multiplied by the mean or median follow up in each study to obtain the denominator (person-years of follow-up) and the number of SCD events contributed to the numerator. Incidence rates were expressed as per 100 person-years.

Summary estimates of incidence and incidence rates of SCD were obtained using a DerSimonian and Laird random effects model [12]. The percentage of variability across studies due to heterogeneity beyond chance was estimated using I^2^ statistics [13] and Cochran’s Q tests of homogeneity. We explored sources of heterogeneity across the estimates of SCD cumulative incidence using univariate meta-regression [13] and subgroup analysis by comparing summary results obtained from subsets of studies dichotomized based on study mean age, proportion of hypertensive patients, proportion of diabetic patients, follow-up duration, study mean BMI, study mean systolic blood pressure, study mean diastolic blood pressure, method of SCD ascertainment (chart review vs. rigorous assessment), and type of cohort (general population vs. selective). We classified studies as a general population cohort if the study encompassed a wide spectrum of patients on dialysis with a broad eligibility criterion and as a selective cohort if the study aimed to capture a subset of patients on dialysis. A two-sided *p*-value <0.05 was considered statistically significant for all analyses. All statistical analyses were performed with Stata/IC version 12.0 (Stata Corp., College Station, TX, USA).

## Results

### Study and included patient characteristics

**Fig. 1:**
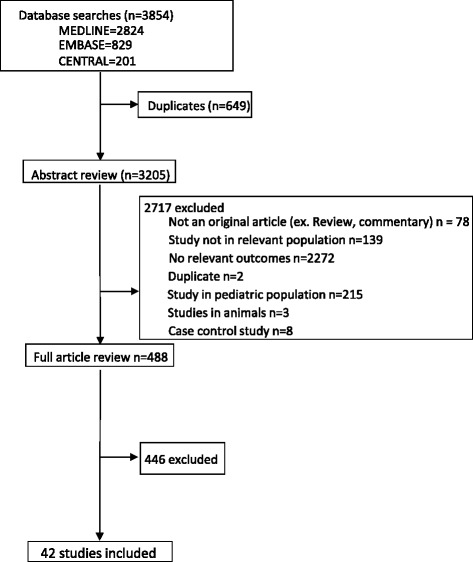
PRISMA flow diagram showing the identification process for eligible studies

The literature search yielded a total of 3,854 citations, of which 488 qualified for full text review (Fig. 1). The final analysis included 42 studies with 80,382 patients with ESRD reporting on 8,574 SCD events. The characteristics of the studies included in the systematic review are shown in Additional file 2. Of the 42 studies, 40 were cohort studies, 1 was a randomized controlled trial, and 2 were observational analyses of randomized controlled trials. 14 were from Asia [14–27], 17 from Europe [28–44], 7 from North America [30, 45–52], 1 from Oceania [53], 2 from South America [54, 55], and 1 international study conducted across centers in Europe, North America and Oceania [56]. The studies were published between 1985 and 2014 and the number of patients enrolled ranged from 22 to 37,765. Most studies were conducted in hemodialysis patients (71 %) with only two enrolling specifically peritoneal dialysis patients [27, 38]. The mean age of patients ranged from 44 to 71 years and the proportion of males ranged from 48 to 83 %. Data on comorbidities were generally limited. The proportion of patients with hypertension across the included studies ranged between 5 and 91 % while those with diabetes ranged between 6 and 100 %.

### Study quality

**Fig. 2:**
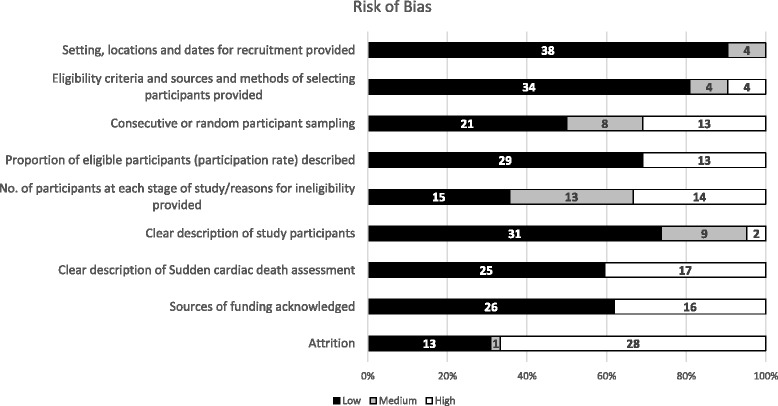
Risk of Bias Assessment

The assessment of the risk of bias in the included studies is summarized in Fig. 2. Thirty-eight studies provided information regarding the setting, locations and dates of studies [14–19, 21–37, 40–43, 45–48, 51–57]. Furthermore, 34 of 42 studies (81 %) had clearly defined eligibility criteria [14, 15, 17–19, 21, 24–31, 33–38, 40–44, 46–48, 51–57]. Twenty one (50 %) studies had a low risk of selection bias based on their random or consecutive sampling method [15, 21, 24, 26, 29–31, 34–36, 41–43, 46, 48, 51, 52, 54, 55, 57]. The eligibility criteria and participant rate were described adequately in 29 studies (69 %) [14, 15, 19, 21, 24–31, 33–35, 37, 41–44, 46–48, 51–55, 57], and 15 studies (36 %) [19, 21, 24, 28–31, 35, 37, 42, 44, 47, 48, 54, 55] described the flow of participants and reasons of ineligibility. Thirty one studies (74 %) provided a clear description of the characteristics of the included participants [14, 15, 17–19, 21, 24, 26–35, 37, 41–46, 48, 51–56] and 24 studies (57 %) appropriately defined sudden cardiac death [15, 17, 19–21, 23, 27, 29, 31, 33–37, 41, 42, 46, 51–57]. Additionally, 26 and 13 studies acknowledged the sources of funding [14, 16, 18, 21, 23, 24, 26–29, 31–34, 36, 38, 41–43, 45, 47, 51–54, 56] and attrition [17–19, 21, 24, 27, 29, 31, 35, 37, 48, 51, 53, 55], respectively.

Of the three studies with data from clinical trials, the overall risk of bias was low. Although only one study provided details about allocation bias [32], the risk of bias associated with blinding, randomization and selective outcome reporting was low.

### Definition of SCD and SCD assessment

The definition of SCD varied among the included studies. Twenty-six (of which 3 were from trials) of the 43 studies provided a definition for SCD [15, 17–21, 23, 27, 29, 31, 32, 34–37, 41, 42, 46, 48, 51–54, 56, 57]. Seventeen studies included time in their definition of SCD [15, 17, 19–21, 23, 27, 29, 31, 34–36, 41, 42, 46, 52, 54]. Eight studies included hyperkalemia, cardiac arrhythmia and cardiac arrest in their definitions of SCD [21, 32, 44, 48, 51–53, 56] and one study broadened their definition to include any unwitnessed and unexpected cardiac death [52]. The most common definitions of SCD were “death occurring within an hour of symptom onset with no clinical support for another cause” (*n* = 12; 28 %) where SCD incidence ranged from 0.76 to 7.09 SCD events per 100 person years and “death occurring within 24 h of symptom onset with no clinical support for another cause” (*n* = 5; 12 %) where SCD incidence ranged from 2.09 to 3.38 SCD events per 100 person-years.

Similar to the variability in the definition of cardiac death, the rigor of assessment of SCD varied among the included studies. Seven studies did not provide any information about the assessment of SCD [14, 23, 33, 38, 43, 47, 49]. Nineteen studies assessed SCD using death certificates and chart reviews [15, 16, 18, 20, 24, 25, 28–32, 34–36, 41, 44, 48, 52, 57] and 16 studies assessed SCD using a more rigorous method, i.e., using blinded assessment, physician interviews, coroner’s report and/or witness interviews [17, 19, 21, 22, 24, 26, 27, 35, 37, 40, 42, 45, 46, 50, 51, 53–56].

### Estimated incidence rates of sudden cardiac death

**Fig. 3:**
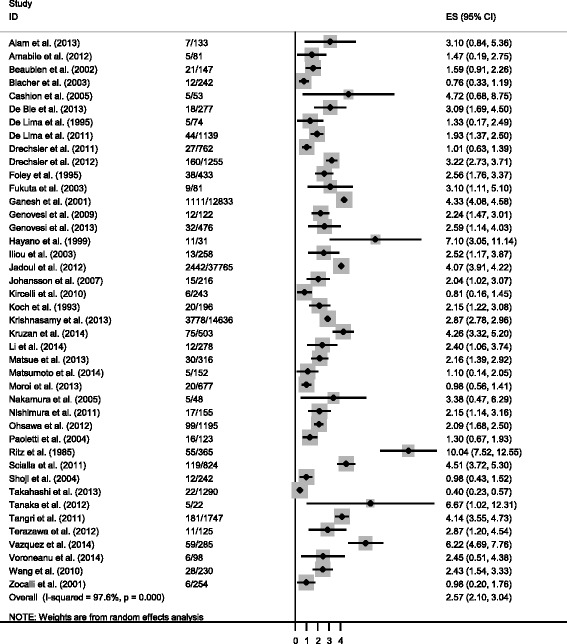
Estimated incidence rate

A total of 8,574 SCD events occurred in 80,382 participants over a follow-up that ranged between 1.5 and 10 years. The estimated incidence rate of SCD among adults with ESRD ranged from 0.4 to 10.04 deaths per 100 person-years (Fig. 3). We observed evidence of significant heterogeneity across the included studies (I^2^ = 98 %, *p* < 0.001) and therefore did not calculate a summative estimate of the incidence rates. Additionally, the incidence rates were not significantly different between the two most common definitions of SCD (*p* = 0.1). The incidence rate in studies that included time in their definition was 2.98 SCD per 100 person-years (95 % CI: 2.87–3.11) and was 2.66 SCD per 100 person-years 95 % CI: 2.57–2.76) for studies that included hyperkalemia in their definition.

### Subgroup analysis

**Fig. 4:**
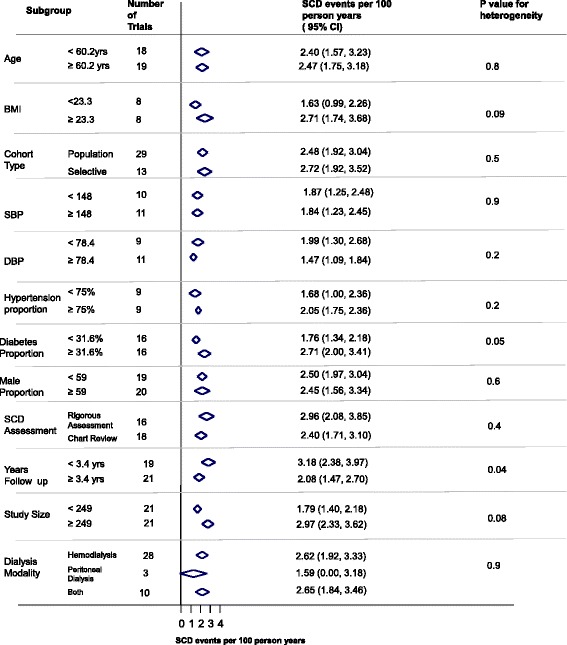
Subgroup analysis

Studies with greater than or equal to 32 % of subjects with diabetes had a greater incidence of SCD compared to studies with less than 32 % of subjects with diabetes (*p* = 0.05; Fig. 4). Studies that had greater than or equal to 3.4 years of follow up had a lower estimated incidence rate of SCD compared to studies with less than 3.4 years of follow up (*p* = 0.04; Fig. 4).

## Discussion

Our quantitative review of randomized controlled trials and cohort studies including over 81,000 patients with ESRD on dialysis shows that the reported incidence of SCD in ESRD varies widely, ranging from 0.4 to 10.04 deaths per 100 person-years. In addition, there is a lack of standardization in the definitions of SCD in ESRD as well as the methods of its assessment.

The lack of standardization and variability in SCD definition has previously been identified in the general population [58]. We have identified similar heterogeneity in SCD definition among dialysis patients as well as inconsistencies in adjudication methods.

The current lack of a standardized definition of SCD in ESRD, in addition to the varied methods of its assessment, are likely to be major contributors to the wide range of estimates reported. As was the case with the systematic review conducted in the general population [58], studies with the primary aim of determining the incidence of SCD was lacking as we observed only 1 study specifically focused on the assessment of the incidence of SCD.

Large, population-based renal registries such as the United States Renal Data System (USRDS) and the Australia and New Zealand Dialysis and Transplant (ANZDATA) Registry have reported causes of death, including SCD (cardiac arrest), among dialysis patients. While large-scale multicenter studies are currently needed to further validate results derived from these registries, it is reassuring that our results are largely consistent with registry-based SCD the incidence rate reported by the United States Renal Data System (USRDS) (4.3 events per 100 person-years) [59] and the proportion of cardiac death as reported by The Australia and New Zealand Dialysis and Transplant Registry (ANZDATA) (2.3 %) [60].

Our subgroup analysis found that studies with higher proportion of patients with diabetes reported a higher incidence of SCD. Diabetes has been found to increase SCD risk in both healthy subjects and subjects with CKD. Indeed, the ‘Paris Prospective Study I’ , a 23-year long prospective study of 7,000 males suggested that diabetes was associated with a SCD risk ratio of 2.2, higher than any other factor evaluated in the study [61]. Moreover, an analysis of 400 SCD cases by Karnik et al. in a cohort of hemodialysis patients found that patients who died of SCD were more likely to have diabetes compared to patients who died of other causes (61.8 vs. 46.8 %) [62]. Finally, we found that studies with a longer follow-up time had a higher estimated incidence of SCD compared to those with a shorter follow-up time. While there were no apparent differences in comorbidities or SCD assessment between these studies, one possible explanation could be a higher chance of loss to follow-up in longer studies compared to shorter studies. This, however, cannot be confirmed as many studies did not report on attrition.

Our systematic review has limitations. Our analysis was restricted by the overall paucity of studies reporting on SCD incidence and was based on published study-level data. With only two studies reporting incidence rates, we were limited in our ability to assess the incidence of SCD in patients on dialysis. We were limited in our ability to explore the effects of race on SCD incidence rate through subgroup analyses as only 6 of the 43 included studies reported on race of the subjects. Another limitation of this study is the lack of inclusion of all large registry data. While the primary purpose of this study was to summarize data from published studies, large national registry databases provide important information with regards to SCD in the dialysis population and future studies should focus on verifying and improving the accuracy of these databases with large validations studies designed to assess the incidence of SCD in patients with ESRD. This study highlights the need for the standardization of SCD definition and ascertainment, and for larger multicenter studies that aim to determine the incidence of SCD in this high risk population.

## Conclusion

The reported incidence of SCD in patients with ESRD varies widely and studies specifically designed to determine the incidence of SCD in adults with ESRD are limited. As SCD remains a major concern in the patients with ESRD our findings emphasize that further investigation to determine the incidence of SCD within this patient population is needed.

## Abbreviations

ESRD, end stage renal disease; SCD, sudden cardiac death

## Additional files

Additional file 1: Includes details regarding the search strategy used for the systematic review. (DOC 28 kb) Additional file 2: Includes a table describing the characteristics of the included studies. (DOC 96 kb)
