# Supplementary material for: Incidence of sudden cardiac death in adults with end-stage renal disease: a systematic review and meta-analysis
Source: BMC Nephrol. 2016 Jul 11;17:78. doi: 10.1186/s12882-016-0293-8 (PMC4940956; doi:10.1186/s12882-016-0293-8)
Supplement: Additional file 1: — Includes details regarding the search strategy used for the systematic review. (DOC 28 kb) [file 12882_2016_293_MOESM1_ESM.doc]

**Supplementary file 1: Search strategy**

MEDLINE and EMBASE

1. exp Randomized Controlled Trial/

2. exp cohort studies/

3. 1 or 2

4. exp renal insufficiency Chronic/

5. exp renal replacement therapy/

6. kidney failure.tw.

7. dialysis.tw.

8. (end-stage renal or end-stage kidney or endstage renal or endstage kidney).tw.

9. (ESRF or ESKF or ESRD or ESKD).tw.

10. or/3-9

11. (hemodialysis or haemodialysis).tw.

12. exp Heart Arrest/

13. (((sudden adj3 cardiac adj3 death*) or sudden) adj3 cardiac adj3 arrest*).tw.

14. sudden death.tw.

15. cardiac.tw.

16. cardiac disease$.tw.

17. cardiac arrest.tw.

18. or/10-17

19. 3 and 10 and 18

20. limit 19 to humans

Cochrane CENTRAL

1. exp renal insufficiency Chronic/

2. exp renal replacement therapy/

3. kidney failure.tw.

4. dialysis.tw.

5. (end-stage renal or end-stage kidney or endstage renal or endstage kidney).tw.

6. (ESRF or ESKF or ESRD or ESKD).tw.

7. or/1-6

8. (hemodialysis or haemodialysis).tw.

9. exp Heart Arrest/

10. (((sudden adj3 cardiac adj3 death*) or sudden) adj3 cardiac adj3 arrest*).tw.

11. sudden death.tw.

12. cardiac.tw.

13. cardiac disease$.tw.

14. cardiac arrest.tw.

15. or/8-14

16. 7 and 15
