# Supplementary material for: Incidence of sudden cardiac death in adults with end-stage renal disease: a systematic review and meta-analysis
Source: BMC Nephrol. 2016 Jul 11;17:78. doi: 10.1186/s12882-016-0293-8 (PMC4940956; doi:10.1186/s12882-016-0293-8)
Supplement: Additional file 2: — Includes a table describing the characteristics of the included studies. (DOC 96 kb) [file 12882_2016_293_MOESM2_ESM.doc]

**Supplementary file 2: Characteristics of the included studies**

| **Study (year of publication)** | **Inclusion criteria** | **Country** | **Follow up (mean years)** | **Number of participants** | **Mean age (years)** | **Male (%)** | **Diabetes (%)** | **Hypertension (%)** | **Definition of SCD** | **Number of SCD events** |
| --- | --- | --- | --- | --- | --- | --- | --- | --- | --- | --- |
| Alam et al. (2013) | Adult patients receiving chronic HD. | Canada | 1.7* | 133 | 66.9 | 60 | 43 | 77 | No definition provided | 7 |
| Amabile et al (2012) | Patients with (i) HD therapy for 3 months, (ii) no clinical cardiovascular complication (myocardial infarction, stroke, emergent coronary revascularization, severe peripheral artery disease requiring revascularization and/or amputation) during the 6-month period preceding entry and (iii) patients agreed to participate in the follow-up study. | France | 4.2* | 81 | 59 | 63 | 48 |  | No definition provided | 5 |
| Beaubien et al. (2002) | Adult patients requiring ongoing dialysis (3 months), and with a technically satisfactory ECG (QT interval measurable from at least eight leads) within 1 month before starting dialysis. | Canada | 9 | 147 |  | 62 |  |  | Death within 1 hour of documented ventricular tachyarrhythmia or in cases of unwitnessed, unexpected death without clinical or post-mortem evidence to support another cause. |  |
| Blacher et al. (2003) | Patients who were on HD for at least 3 months and had no clinical cardiovascular disease during 6 months preceding entry into the study. | France | 6.5 | 242 | 51 | 62 |  |  | Witnessed death that occurred within 1 hour after the onset of acute symptoms, with no evidence that violence or accident played any role in the fatal outcome. | 12 |
| Cashion et al (2005) | Adult patients with ESRD on HD. Patients without atrial fibrillation, an implantable cardiovascular defibrillator and/or a pacemaker were included. | USA | 2 | 53 | 48 | 49 | 25 | 91 | No definition provided | 5 |
| de Bie et al (2013) | All consecutive patients who initiated HD or PD therapy in the university hospitals of Leiden and Amsterdam between January 2000 and December 2009 and remained on dialysis for >3 months. | The Netherlands | 2.1 | 277 | 56 | 62 | 32 | 73 | A subject apparently well, is observed to have died within a few minutes from the onset of symptoms and if the cause of death cannot reasonably be attributed to some potentially lethal disease; (ii) if the patient was found dead after an unwitnessed event with no other cause of death identifiable and if the patient had been in his or her usual state of health without any symptoms when last observed | 18 |
| De Lima et al. (1995) | Adult HD patients in a dialysis center at Sao Paulo, Brazil. | Brazil | 5.1 | 74 | 44.2 | 54 |  |  | No definition provided | 5 |
| De Lima et al. (2011) | Adult kidney transplant candidates on dialysis in the Sao Paulo’s waiting list. | Brazil | 2 | 1139 | 53 | 61 | 5 | 85 | Death from natural causes occurring within 1 h after initiation of symptoms with no definitive assessment of its cause. | 44 |
| Drechsler et al. (2011) | The Netherlands Cooperative Study on the Adequacy of Dialysis (NECOSAD) is an observational prospective follow-up study in which incident dialysis patients have been enrolled in 38 participating dialysis centres since 1997 in the Netherlands. | The Netherlands | 3 (total) | 762 | 59 | 61 | 20 |  | No definition provided | 27 |
| Drechsler et al. (2012) | Adult patients with type 2 diabetes mellitus, aged 18-80 years, and receiving hemodialysis for less than 2 years. | Germany | 4 (mean) | 1255 | 66 | 54 | 100 |  | Patients who died unexpectedly and did not present with serum potassium >7.5 mmol/L before the start of the three most recent sessions of HD | 160 |
| Foley et al. (1995) | Patients on renal replacement therapy in 3 centers in Montreal, Canada and St. John's, Canada. | Canada | 3.4 | 433 | 51 | 64 | 26.8 | 29.1 | No definition provided | 38 |
| Fukuta et al. (2003) | Patients with ESRD on regular HD therapy (a 4-hour hemodialysis session, three times a week) who underwent an elective coronary angiography between 1989 and 1996 for assessment of their CAD. All patients had symptoms and/or clinical signs of CAD, including typical anginal pain, positive exercise electrocardiogram (ECG), and previous history of myocardial infarction. | Japan | 2.6 | 81 | 61.3 | 67 | 35.8 |  | No definition provided | 9 |
| Ganesh et al. (2001) | Randomly selected patients with prevalent HD receiving thrice weekly HD treatment from 2 USRDS datasets for whom complete data was available. | USA | 2 | 12833 | 58 | 50 | 39 |  | When cardiac death and/or hyperkalemia was identified as the cause of death on the notification form. | 1111 |
| Genovesi et al. (2009) | Patients who were on HD from five dialysis centers in Lombardia, Italy. All patients treated in the participating centers in June of 2003 were included. | Italy | 3 | 476 |  | 58 | 19 | 81 | An unexpected natural death occuring within an hour of symptom onset. | 32 |
| Genovesi et al. (2013) | Patients on chronic HD treatment at the San Gerardo Hospital (Monza, Italy) from January 2005 to October 2010. | Italy | 3.8 | 122 | 71.3* | 65 | 27.1 | 84.4 | An unexpected death occurring within an hour of the onset of symptoms | 12 |
| Hayano et al. (1999) | Patients on chronic HD therapy (3 times a week) who underwent elective diagnostic coronary angiography due to symptoms and/or signs suggesting coronary artery disease. | Japan | 5 | 31 | 56 | 74 | 26 | 48 | Sudden death was defined as unexpected or natural death that occurred within 24h of new or more serious symptoms or during sleep or while unobserved | 11 |
| Iliou et al. (2003) | All consecutive patients older than 18 years who attended 1 of 3 Parisian hemodialysis centers between April 12 and 26, 1999 | France | 2 | 258 | 60.2 | 58.1 | 19 |  | Death was considered to be sudden if it occurred in the first hour after the onset of symptoms and to be cardiac if no other evident causes were found. | 13 |
| Jadoul et al. (2012) | Patients on HD who were greater than 17 years of age. | France, Germany, Italy, Spain, United Kingdom, Japan, United states of America, New Zealand, Canada, Belgium and Sweden | 1.6* | 37,765 | 61.8 | 57 | 38 | 90 | Sudden cardiac death was assigned if the primary cause of death was reported as death due to cardiac arrhythmia, cardiac arrest and/or hyperkalemia | 2442 |
| Johansson et al. (2007) | Patients on HD or PD. | Sweden | 3.4* | 216 | 59 | 64 | 31 |  | A natural death occurring within an hour of symptom onset | 15 |
| Kircelli et al. (2010) | Patients on CAPD in a centre in Turkey. | Turkey |  | 243 | 44.1 | 57 | 12.3 | 9.1 | No definition provided | 6 |
| Koch et al. (1993) | Adult HD patients with diabetes. | Germany | 4.75 | 196 |  | 49.5 | 100 | 88 | Cardiac death without other known cause | 20 |
| Krishnasamy et al. (2013) | All adult patients with end-stage kidney failure receiving maintenance dialysis in Australia and New Zealand who died between January 1, 1999, and December 31, 2008. | Australia and New Zealand | 9 | 14,636 | 67.25 | 57.6 |  |  | Cardiac arrest (cause uncertain) and myocardial ischemia (presumed) were the codes for sudden death | 3,778 |
| Kruzan et al. (2014) | Dialysis patients, the Choices for Healthy Outcomes in Caring for ESRD (CHOICE) Study. | USA | 3.5 | 503 | 58 | 54 |  |  | No definition provided | 75 |
| Li et al. (2014) | HD patients (HD for[3 months) at Guangdong General Hospital (GGH) Renal Division (a Center Of Kidney Disease Care in Southern China and the Hemodialysis Quality Control Center in Guangdong Province) between April 2009 and April 2011. | China | 1.8 | 278 |  | 53.6 | 33.8 | 26.6 | No definition provided | 12 |
| Matsue et al. (2013) | Patients >18 years old on HD for >3 years. | Japan | 4.4 | 316 | 65.2 | 66 | 44 | 37 | An abrupt, unexpected death within 1 hour symptom onset OR autopsy findings consistent with SCD | 30 |
| Matsumoto et al. (2014) | Patients undergoing first coronary revascularization in 26 centers in Japan from January 2005 through December 2007. | Japan | 3 | 152 | 67.7 | 59 | 31 | 4.6 | No definition provided | 5 |
| Moroi, M. et al. (2013) | Age>20 years with at least 1 CV risk factors (hypertension, diabetes melitus, hypercholesteremia, peripheral artery disease, history of congestive heart failure, hypotension). | Japan | 3.2* | 677 | 63.6 | 60 | 37.4 | 86 | Cause of death that could not be determined within 24 hours of occurrence | 20 |
| Nakamura et al. (2005) | HD patients who were suffering from cardiac symptoms such as congestive heart failure, ventricular arrhythmia, or chest pain, and who had been admitted to our hospital (National Cardiovascular Center) between April 1994 and April 1997 for the diagnosis and treatment of cardiovascular diseases. | Japan | 3.1 | 48 | 61 | 83 | 27 |  | Sudden death was defined as a witnessed death that occurred within 1 h after the onset of symptoms with no history of violence or accident playing any role in the fatal outcome. | 5 |
| Nishimura et al. (2011) | Patients on HD who did not have significant stenotic or obstructive coronary lesions. | Japan | 5.1 | 155 | 63.7 | 57 | 559 |  | Death within 24 h of the time the patient was last seen alive in a normal state of health and for which cardiac diseases such as malignant arrhythmias or acute coronary syndrome were the cause. | 17 |
| Ohsawa, M. et al. (2012) | Patients on HD | Japan | 3.9 | 1195 | 61 | 64 |  |  | No definition provided | 99 |
| Paoletti, E., et al. (2004) | Patients on HD for 6 months | Italy | 10 | 123 | 63.1 | 62 | 20.3 |  | No definition provided | 16 |
| Ritz et al. (1985) | All diabetic patients who entered uremia treatment programs in 17 German dialysis centers between January 1, 1972 and December 31, 1983 and who were recorded on the EDTA registry were entered into the study. | Germany | 1.5 | 365 |  |  |  |  | Death/cardiac arrest due to unknown cause in less than 1 hour | 55 |
| Scialla, J. J. et al. (2011) | Patients initiating HD | USA | 3.2* | 824 | 57.1 | 53 | 55.8 |  | Sudden cardiac death was defined as an out-of-hospital death with the following codes: ICD-9 390–398,402 or 404–429; and ICD-10, I00-I09, I11, I13 and I20–I51, as described previously | 119 |
| Shoji et al. (2004) | HD patients, approved by the ethical committee at Inoue Hospital, Japan. | Japan | 5.1 | 242 | 56 | 48 | 24.5 |  | Sudden death was defined as a witnessed death that occurred within 1 h after the onset of acute symptoms, with no evidence of accident or violence. | 12 |
| Takahashi et al. (2013) | Patients receiving HD therapy in Nagoya Kyoritsu Hospital, Kaikokai Central Clinic, Meiko Kyoritsu Clinic,Ama Kyoritsu Clinic, Anjo Kyoritsu Clinic, and Hekikai Kyoritsu Clinic, in Japan, between November 2000 and December 2007. | Japan | 4.2 | 1290 | 61 | 64 |  | 71.9 | No definition provided | 22 |
| Tanaka, S., et al. (2012) | Adult HD patients who underwent elective coronary angiography and demonstrated both stress-induced myocardial ischemia and significant coronary artery stenosis | Japan | 3.42 | 22 | 67 | 64 | 64 |  | No definition provided | 5 |
| Tangri et al. (2011) | Prospective randomized multicenter clinical trial that enrolled long-term HD patients at 15 clinical centers composed of 72 dialysis units in the USA | USA | 2.5 | 1747 |  |  |  |  | Sudden death was defined as a witnessed or unwitnessed unexpected death with preceding duration of symptoms less than 24 hours for witnessed deaths and less than the interval since the last dialysis session for unwitnessed deaths. Sudden death was considered SCD if the patient had a history of IHD, arrhythmias, or presence of other heart disease in the absence of IHD or arrhythmias. | 181 |
| Terazawa et al. (2012) | Patients who underwent a coronary artery bypass graft or percutaneous cornary intervention using drug-eluting stents and have ESRD requiring dialysis. | Japan | 3.1 | 125 | 64.3 | 77 | 58.4 | 77.6 | No definition provided | 11 |
| Vazquez et al. (2014) | Patients who began dialysis (hemodialysis or peritoneal dialysis) in Complejo Hospitalario de Jaén, Spain and its dependent dialysis centers between November 1, 2003 and September 15, 2007. | Spain | 3.3 | 285 | 66 | 55 |  |  | (i) SD at 24 h (SD 24H): unexpected death that occurred within 24 h following the start of the symptoms, or when the patient was found dead but had been observed to be alive 24 h earlier; (ii) SD at 1 h (SD 1H): death witnessed as having occurred in the first hour following the start of symptoms; (iii) SD in dialysis: unexpected death during a session of hemodialysis, and (iv) SD at weekend: death occurring after 48 h from the last dialysis session. | 59 |
| Voroneanu et al. (2014) | Patients undergoing chronic HD treatment for at least 3 months in a single unit were assessed for eligibility for inclusion. | Romania | 2.5 | 98 | 55.4 | 50 | 6.1 | 67.3 | No definition provided | 6 |
| Wang, A. Y, et al. (2010) | Adults with ESRD on continuous long term PD | China | 5 | 230 | 56 | 51 | 30 |  | Sudden unexpected natural death within 1 hour from the symptom onset and without any immediate previous condition that could appear fatal | 28 |
| Zocalli et al. (2001) | Patients who have been on regular dialysis treatment for at least 6 mo, with LV ejection fraction 35% and without a history of clinical evidence of circulatory congestion. | Italy | 2.4 | 254 | 60.3 | 57 | 15 |  | No definition provided | 6 |
